# Habitat detection, habitat choice copying or mating benefits: What drives conspecific attraction in a nomadic songbird?

**DOI:** 10.1111/1365-2656.13844

**Published:** 2022-11-22

**Authors:** Shannon Buckley Luepold, Hanna Kokko, Alex Grendelmeier, Gilberto Pasinelli

**Affiliations:** ^1^ Swiss Ornithological Institute Sempach Switzerland; ^2^ Department of Evolutionary Biology and Environmental Studies University of Zürich Zürich Switzerland; ^3^ Organismal and Evolutionary Biology Research Program, Faculty of Biological and Environmental Sciences University of Helsinki Helsinki Finland

**Keywords:** conspecific attraction, decisions, habitat selection, hidden lek, settlement, social information, songbirds, territory clustering

## Abstract

1. Conspecific attraction during habitat selection is common among animals, but the ultimate (i.e. fitness‐related) reasons for this behaviour often remain enigmatic.
2. We aimed to evaluate the following three hypotheses for conspecific attraction during the breeding season in male Wood Warblers (*Phylloscopus sibilatrix*): the habitat detection hypothesis, the habitat choice copying hypothesis and the female preference hypothesis. These hypotheses make different predictions with respect to the relative importance of social and nonsocial information during habitat assessment, and whether benefits accrue as a consequence of aggregation.
3. We tested the above hypotheses using a combination of a 2‐year playback experiment, spatial statistics and mate choice models.
4. The habitat detection hypothesis was the most likely explanation for conspecific attraction and aggregation in male Wood Warblers, based on the following results: (1) males were attracted to conspecific song playbacks, but fine‐scale habitat heterogeneity was the better predictor of spatial patterns in the density of settling males; (2) male pairing success did not increase, but instead slightly decreased, as connectivity with other males (i.e. the number and proximity of neighbouring males) increased.
5. Our study highlights how consideration of the process by which animals detect and assess habitat, together with the potential fitness consequences of resulting aggregations, are important for understanding conspecific attraction and spatially clustered distributions.

## Graphical abstract

The simplest hypothesis for conspecific attraction, that conspecifics inform others that potential habitat is present, is overlooked in many studies. Recent work suggests that this mechanism is the likely explanation for conspecific attraction in male wood warblers, and underscores the importance of considering the hierarchical nature of the settlement process.

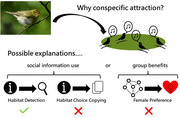

## 1 INTRODUCTION

Patterns in the spatial distribution of many animals emerge from habitat selection by individuals. The tendency to settle near conspecifics (‘conspecific attraction’) is taxonomically widespread, but tests of the putative fitness benefits driving this behaviour are relatively rare (Buxton et al., 2020). There are at least three nonmutually exclusive reasons why an animal might settle in a location where conspecifics are present (Stamps, 2001): (1) the presence of conspecifics increases the detectability of potentially suitable habitat (thereby reducing search costs); (2) copying the choices of conspecifics reduces costs of or errors in habitat evaluation; (3) proximity to conspecifics increases fitness after settlement (Allee effects).

The first mechanism (that conspecific presence increases the detectability of potentially suitable habitat) is the simplest of the three and can be viewed as a null hypothesis for conspecific attraction. This is because conspecific attraction is to some extent always a product of increased detection probability—animals must become aware that potentially suitable habitat is present before they can assess it and make a choice, and a habitat patch containing conspecifics is almost certainly more readily detectable (via sound, pheromones, etc.) than an empty patch of habitat (Fletcher, 2006). The habitat detection hypothesis posits that conspecific presence informs individuals seeking somewhere to settle that the local habitat *may* be suitable, not that it *is* suitable. In other words, social information (i.e. information acquired from observing or interacting with others, Wagner & Danchin, 2010) is most relevant up until arrival in a habitat, at which point the choice to remain or not is based on an individual's own (presumably accurate) perception of the environment (i.e. nonsocial information, sensu Wagner & Danchin, 2010).

Alternatively, the presence of conspecifics may be a cue to arriving individuals that the local habitat *is* suitable and thus the decisions of settled conspecifics are copied (habitat choice copying hypothesis). As copying is by definition imitative, it requires prioritization of social information over nonsocial information derived from independent assessment. The suggestion that social information is prioritized over nonsocial information during habitat evaluation is widespread in the literature on breeding habitat selection in birds (e.g. Betts et al., 2008; Forsman et al., 2008; Nocera et al., 2009; Valente et al., 2021), where it is often argued that copying the choices of others is adaptive.

The putative benefits of using social information to detect or evaluate habitat (reduced search time and more efficient or accurate habitat assessment respectively) are difficult to test in wild animals, because one cannot know how long settlers would have searched, or what an alternative habitat choice would have been, had they not encountered conspecifics. Nevertheless, the habitat detection hypothesis and the habitat choice copying hypothesis differ in their prediction of whether social or nonsocial information is prioritized during habitat assessment, and hence which is a more important predictor of settlement patterns (Table 1).

**TABLE 1:** Predictions of three hypotheses for conspecific attraction and territory aggregation in male songbirds

	Predictions	
Hypothesis	1. Within a habitat patch containing conspecific song playbacks, the more important predictor of ♂ settlement density is:	2. The effect of being close to other ♂s on ♂ pairing success is:
Habitat Detection	Spatial variation in habitat attractiveness^a^ (with higher densities in more attractive habitat)	No prediction
Habitat Choice Copying	Proximity to conspecific playbacks of any type or to those with low song rates (with higher densities closer to playbacks)	No prediction
♀ Preference	No prediction	Positive

^a^ Spatial variation in habitat attractiveness = spatial variation in cumulative nest densities (see text for details).

Crucially, using conspecifics as cues during habitat search or assessment may lead to outcomes where individuals reside closer to each other than would be ideal in terms of competition avoidance. In such situations, competition to some extent erodes the original benefit(s) of increased search efficiency and/or location of superior resources (Childress & Herrnkind, 2001; Stamps & Krishnan, 2005). Individuals may try to mitigate the costs of intraspecific competition by, for example, settling near conspecifics of relatively low competitive ability. For example, Szymkowiak et al. (2016) reported in an experimental playback study that Wood Warblers (*Phylloscopus sibilatrix*) preferentially settled near playbacks with low song rate (representing males of apparently low competitive ability). Szymkowiak et al. (2016) suggested that selectively settling near individuals based on their apparent competitive ability was essentially a ‘compromise’ resulting from reliance on social information and the need to avoid potentially dominant competitors.

In contrast, there are several hypotheses predicated on the idea that attracted individuals derive postsettlement social benefits (Safran et al., 2007; Stamps, 1988). For example, male aggregations during the breeding season are often attributed to mate attraction benefits (Fletcher & Miller, 2006; Höglund & Alatalo, 1995). For lekking species as well as some territorial songbirds, a frequently cited mechanism leading to the evolution of aggregative behaviour in males is female preference for appraising males in groups (Bradbury, 1981; Kokko, 1997; Wagner, 1998). According to the female preference hypothesis, per capita mating success should be higher for aggregated males because solitary males are largely ignored by females. While the female preference hypothesis predicts male conspecific attraction and aggregation, it does not make specific predictions about the relative importance of social versus nonsocial information for male settlement decisions. That is, aggregated males might obtain mating benefits regardless of whether facilitated habitat detection or habitat choice copying contribute to forming the initial aggregation.

We combined a playback experiment, spatial statistics and mate choice models to evaluate whether habitat detection, habitat choice copying or postsettlement mating benefits could explain male conspecific attraction in the Wood Warbler. The Wood Warbler is a migratory passerine in which territory aggregations occur (Broughton et al., 2020; Herremans, 1993) and male conspecific attraction has been demonstrated experimentally (Grendelmeier et al., 2017; Szymkowiak et al., 2016). Low breeding site fidelity coupled with large interannual fluctuations in local population sizes has led to the characterization of Wood Warblers as ‘nomadic’ (Teitelbaum & Mueller, 2019; Wesołowski et al., 2009), and movements within a breeding season are also frequent (Herremans, 1993; Luepold, 2022; Norman, 1994). Although the benefit of enhanced mate attraction has been hypothesized to be an important contributor to male aggregative behaviour in this species, previous studies have yielded conflicting results with respect to the effect of aggregation on male pairing success (Grendelmeier et al., 2017; Herremans, 1993). There has also been no attempt to disentangle the relative importance of social and nonsocial information in guiding male settlement decisions. We tested two sets of predictions (Table 1) to distinguish between the habitat detection hypothesis, the habitat choice copying hypothesis and the female preference hypothesis.

## 2 METHODS

### 2.1 Study species and study sites

Wood Warblers are Palaearctic, long‐distance migratory songbirds that breed in forests across Eurasia. In the Swiss Jura Mountains, males begin arriving to breeding areas in mid‐April and females start arriving in late April. Females select the nest site and build the nest, which is invariably on the ground (Cramp, 1992). Following nest predation, females will attempt to renest up until the end of June, at which time male singing activity also declines.

**FIGURE 1:**
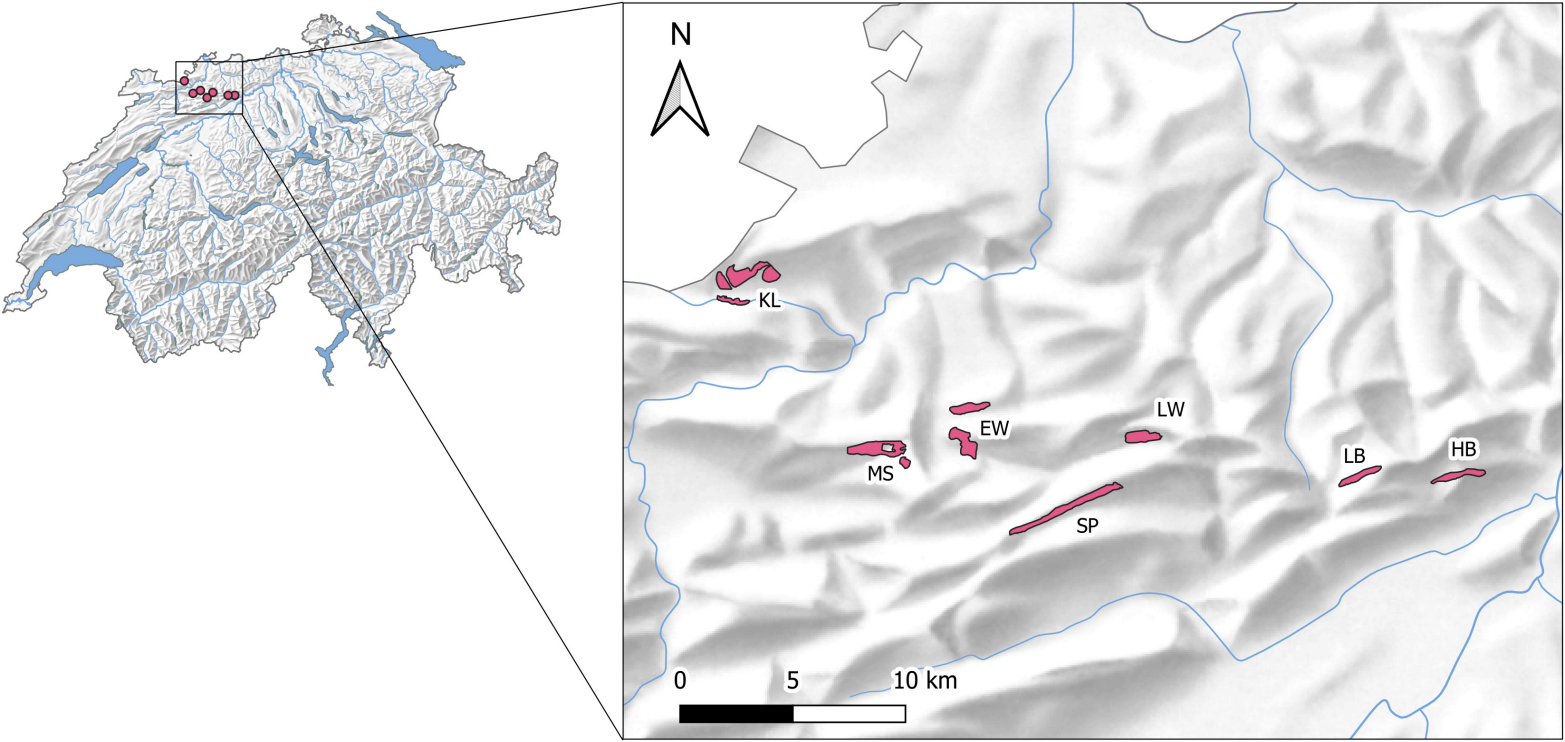
Location of study sites in the Swiss Jura Mountains. Basemap © swisstopo, Bern, Switzerland.

We studied Wood Warbler breeding at seven sites (forests) in the Swiss Jura Mountains between 2010 and 2020 (Figure 1). Some study sites consisted of a single forested hillside (HB, LB, LW, SP), while others consisted of several hillsides in close proximity (EW, KL, MS). Hillsides ranged in size from *c*. 50–190 ha, and in altitude from *c*. 450–1000 m above sea level. The minimum distance between sites was *c*. 2.2 km. European beech (*Fagus sylvatica*) was the predominant tree species on all hillsides except one, where the forest was comprised largely of oak species (*Quercus* sp.) and hazel (*Corylus avellana*).

### 2.2 Data collection

#### 2.2.1 Quantifying settlement patterns and spatial behaviour

From 2010 to 2020, as part of a long‐term research project on the breeding ecology of Swiss Wood Warblers, we searched for and monitored Wood Warbler nests throughout the breeding season (mid‐April until early July). For the purpose of studying conspecific attraction and settlement behaviour, we quantified individual space use and settlement patterns in more detail between 2017 and 2019. To do this, we used a combination of territory mapping (Bibby et al., 2000), radio telemetry and resighting colour‐ringed individuals (*c*. 75% of adults were colour ringed, *n* = 270, see Supporting Information S1 for further details). During our regular (every 1–2 weeks) surveys for territory mapping, we recorded the singing locations (i.e. territories) of all colour‐ringed as well as unmarked individuals within a site. Telemetry and resighting of colour‐ringed birds provided more detailed information on individual space use, including shifts in territory location. By combining these approaches, we obtained a comprehensive picture of settlement dynamics across the breeding season.

#### 2.2.2 Playback experiment

To test the first set of predictions (Table 1), in 2017 and 2018 we conducted an experiment at a subset of the study sites (EW, HB and LB) to compare male settlement patterns in response to playback of conspecific song and spatial variation in habitat features. Based on the suggestion of previous studies in Wood Warblers that high song rates indicate males of high quality or competitive ability (Szymkowiak et al., 2016; Szymkowiak & Kuczyński, 2017), we provided playbacks with both high and low song rates (see below for details).

To create playback song tracks, we used the software Audacity (version 2.1.2) to edit .wav files of singing males recorded in Switzerland, France or Germany between 2010 and 2016. Wood Warblers sing two types of songs: trill songs and piping songs (Cramp, 1992). During any given singing bout, trill songs are generally much more frequent (mean ± SD = 5.07 ± 0.12 songs min^−1^, *n* = 99 males) than piping songs (mean ± SD = 0.90 ± 0.05 songs min^−1^, *n* = 99 males) so we created tracks for playback with one piping song per minute and either three trill songs per minute (representing a male of low competitive ability) or six trill songs per minute (representing a male of high competitive ability). A total song rate of four songs per minute is below average in our population (mean = 5.97 ± 0.14 songs min^−1^, *n* = 99 males), while a song rate of seven songs per minute corresponds to the upper quartile (6.97 songs min^−1^, *n* = 99 males).

We deployed an array of song playback stations representing males of high and low competitive ability within each site: EW in 2017 (4 high, 7 low); LB in 2017 (3 high, 2 low); EW in 2018 (6 high, 7 low), LB in 2018 (2 high, 2 low); HB in 2018 (2 high, 1 low). Sites that were selected for the experiment were known to contain suitable breeding habitat, as indicated by Wood Warbler breeding there at least three times between 2010 and 2016. However, habitat structure within each forest was not homogeneous, and therefore playback stations were necessarily distributed across spatial gradients in habitat features.

To avoid pseudoreplication, each station played the song of a unique male. Playback stations consisted of two speakers (Maxxtro 2.0) mounted *c*. 1 m above‐ground and spaced 100 m apart, alternating broadcasts every 3 min to simulate the movement of a singing individual (see Grendelmeier et al., 2017 for further details). Thus, the midpoint between the two speakers was considered the ‘territory centre’ for a simulated male (see Tables S1–S3 for nearest neighbour distances and overall playback densities per site). ‘High’ and ‘low’ song rates were assigned randomly to playback stations, and locations that were ‘occupied’ in both 2017 and 2018 (i.e. had a playback station) broadcast a different song rate each year (e.g. high song rate in 2017, low song rate in 2018). We did not have a ‘control’ treatment because our aim was not to establish *whether* song playback affects settlement—two previous experiments in Wood Warblers (Grendelmeier et al., 2017; Szymkowiak et al., 2016) have found that it does (i.e. conspecific attraction occurs in this species). Rather, we sought to tease apart the relative importance of social versus nonsocial information for settlement decisions by examining what birds do when conspecific locations are not exactly aligned with fine‐scale spatial variation in preferred habitat features. The relative importance of social versus nonsocial information provides insight into whether attraction is attributable to increased detection probability (followed by independent assessment), or whether it is the result of individuals copying the choices of others. If spatial variation in habitat is the better (i.e. more important) predictor, this suggests that nonsocial information is more important for settlement decisions. If proximity to conspecifics is the better predictor, this suggests that social information is more important (i.e. it is prioritized to the point of blind copying). Previous experiments (Grendelmeier et al., 2017; Stelbrink et al., 2019) found Wood Warblers did not respond to noise control playbacks of Common Wood Pigeon (*Columba palumbus*), demonstrating that attraction to conspecific playback is not attributable merely to the presence of novel, digital sounds.

Songs were broadcast from *c*. April 10 (prior to the arrival of the first males in the study sites) until mid‐June. Following Grendelmeier et al., 2017, songs broadcast 5 days/week (not on Wednesdays and Sundays), beginning at 6:00 and continuing until 16:00. We silenced playbacks 2 days/week in order to reduce habituation. Amplitude was standardized to *c*. 80–90 dB at 1 m above the speaker, and songs were audible up to *c*. 100 m from the speakers. The singing locations of marked and unmarked males were recorded on a regular basis throughout the breeding season (see above and Supporting Information).

All experimental and field data collection protocols were approved by the Federal Office of Environment (FOEN), Switzerland, and the veterinary offices of canton Basel‐Landschaft, Solothurn, Jura and Aargau (permit numbers BL468/25097 and BL468/28642).

### 2.3 Data analysis

#### 2.3.1 Defining spatial variation in habitat features (‘habitat attractiveness’)

Below we describe the rationale for our choice of proxy for spatial variation in habitat features and how we derived it. In Wood Warblers, females choose the nest site, which may or may not lie within the area their mate was initially defending (Cramp, 1992; Wesołowski, 1987). Thus, nest site locations reflect habitat conditions deemed suitable by females. While we do not know which specific features of a habitat females heed most when selecting a nest site, we have observed females prospecting by flying from place to place on the ground, and assume their choices are based on some form of assessment. We therefore used kernel density estimation to generate 10 × 10 m continuous rasters representing cumulative nest density (i.e. including all nest locations 2010–2020) for each site, and used these rasters as proxies for spatial variation in habitat features that are attractive to females. We refer to the variable represented by these rasters as *hab* in the following analyses, and provide further details about how the rasters were created in the Supporting Information (see S2). We emphasize that nest densities are *cumulative across years*, and therefore *hab* does not represent a source of social information in the form of a high density of nests that can be observed at a particular time. In other words, for a male settling in year *t*, *hab* does not reflect spatial variation in the density of nests in year *t*.

Importantly, we note that spatial consistency in territory and/or nest locations across years is not an artefact of individual site fidelity but instead reflects the decisions of multiple settlers (individual return rates are <5% in our study sites and in other regions, e.g. Herremans, 1993; Wesołowski et al., 2009). For several proximate and ultimate reasons, this interannual spatial consistency in settlement locations is most likely due to underlying habitat characteristics rather than social information collected in previous years (see Supporting Information S2 for details).

For the analysis of male settlement patterns in relation to spatial variation in habitat attractiveness and proximity to conspecific song playback stations (see ‘Analysis 1’ below), we note that we did not let nests from the same site and year impact the habitat attractiveness estimation. For example, when analysing the settlement pattern for males in EW in 2017, the habitat attractiveness raster was based on nests in EW from all years except 2017. This approach avoids potential bias that may be caused by females being constrained by the location of their mate (females choose nest sites autonomously, but are obviously required to be in physical contact with the male during mating, which may lead to nonindependence of these two spatial locations). This approach yields slight temporal variations in estimated habitat attractiveness for a given spatial location, because each year excludes a different sets of data points. These fluctuations remain minor: habitat attractiveness values for pixels within a given site were highly correlated between years (EW 2017/2018: Pearson's correlation coefficient *r* = 0.800, 95% CI = 0.794–0.807, *p* < 0.001, *n* = 13,926; LB 2017/2018: *r* = 0.813, 95% CI = 0.804–0.822, *p* < 0.001, *n* = 5106).

#### 2.3.2 Analysis 1: Testing the habitat detection and habitat choice copying hypotheses

We created a point pattern for each combination of experimental site and year, representing the cumulative settlement locations of males (ringed and unringed) within that site and year. We therefore had a total of five point patterns (EW in 2017, EW in 2018, LB in 2017, LB in 2018 and HB in 2018), which when combined represented settlement locations for 50 unique males. Settlement at a given location is often temporary among males in our population, and many males are only present within a site for a couple of weeks or less (Luepold, 2022). We therefore considered males as settled, and included them in the point pattern, if they were present within a site for ≥5 days. Due to polyterritoriality and within‐season dispersal, many males also held multiple territories (defined as singing locations ≥150 m apart) over the course of the breeding season. To ensure that all points within a site were independent, we selected one territory per individual to include in the point pattern. For individuals with multiple territories, we selected either the territory with the longest tenure, or when tenures were the same, we selected the first territory location. Because these patterns were cumulative across the season, they included locations of all individuals that were present at some point between mid‐April and mid‐June, and did not reflect the spatial constellation of individuals present at the same time.

We used Poisson point process modelling (PPM) in the r package spatstat (Baddeley et al., 2015) to determine whether spatial variation in proximity to conspecific playbacks of any type, in proximity to conspecific playbacks with low song rates, or in habitat attractiveness best predicted male settlement patterns within a site. These models estimate the density of a point pattern as a loglinear function of one or more covariates, and take the form.

$$\lambda \left(u\right)=\exp\left(\beta_{0}+\beta_{1}S\left(u\right)\right),$$

where λ(*u*) is the estimated density of points at location *u*, and *S*(*u*) is the value of a covariate *S* at location *u*. *β*_0_ and *β*_1_ represent the intercept term and estimate of a given covariate respectively. For the present analysis, we had three spatial covariates: (1) raster representing spatial variation in habitat attractiveness (*hab*), calculated as described above; (2) raster representing spatial variation in proximity to conspecific playbacks of all types (*prox.all*); (3) raster representing spatial variation in proximity to conspecific playbacks with low song rate (*prox.lc*). Playback proximity rasters were created using the formula *e* ^−*d*/200^, where *d* is the linear distance (in meters) from each pixel to the nearest playback. For further methodological details on the playback proximity rasters, please see Supporting Information S3. In order to estimate spatial variation in point density, PPMs employ numerical quadrature (Baddeley et al., 2015; Renner et al., 2015). This technique involves defining a set of weighted quadrature points at which the density function is evaluated (see Supporting Information S4 for more information on the quadrature scheme used in this analysis). We used R version 4.1.3 for PPM analysis (R Core Team, 2022).

We fit common PPMs to all five point patterns using the spatstat function *mppm*. We used Akaike's information criterion (Akaike, 1974; Burnham & Anderson, 2002) corrected for small sample size (AICc, Hurvich & Tsai, 1989) to determine which of the following candidate models best predicted spatial variation in the density of settling males: (1) null model (no covariates); (2) model including habitat attractiveness covariate (*hab*); (3) model including covariate representing proximity to conspecific playbacks of any type (*prox.all*); (4) model including covariate representing proximity to conspecific playbacks with low song rate (*prox.lc*); (5) model including covariates *hab* + *prox.all*; (6) model including covariates *hab* + *prox.lc*. Models with a ΔAICc ≤ 2 relative to the best‐supported model were considered to also have substantial support.

Based on previous experimental demonstrations of male conspecific attraction in this species (Grendelmeier et al., 2017; Szymkowiak et al., 2016), we expected *β*_prox.all_ and/or *β*_prox.lc_ to be positive. However, a positive effect of proximity to conspecific playbacks on male settlement density is consistent with all three hypotheses, and is therefore uninformative for distinguishing between them. To differentiate between the habitat detection hypothesis and the habitat choice copying hypothesis, we assessed the relative importance of conspecific cues and habitat attractiveness in predicting male density. We did this in two ways. First, we compared candidate models containing different combinations of covariates using AICc. Second, we standardized coefficients using range standardization to compare the relative magnitude of their effects (Grace et al., 2018). We calculated the relative importance of each covariate using the formula exp(*β* × range), where *β* represents the model coefficient and range represents the observed range of values of the covariate (Baddeley et al., 2015). For a given covariate, we used the *β* value from the best‐supported model containing that covariate.

#### 2.3.3 Analysis 2: Testing the female preference hypothesis

To test the prediction that males in aggregations are more attractive to females (Table 1), we analysed the choices of females from all study sites (not only the sites with playbacks) between 2017 and 2019. We first determined the locations of the males available to each female. For this we filtered the territory location data to obtain the male locations that were (1) in the site that she settled, and (2) that were closest in time to her nest initiation date. This yielded a map of males that were present in the site when she settled. We did not exclude already paired males, since polygyny is an infrequent but regular occurrence in Wood Warblers (Cramp, 1992). Female Wood Warblers begin prospecting for nest sites immediately upon arrival to a potential breeding area, and typically begin nest building that same day or the day after (Cramp, 1992). On occasion, we were present when females apparently arrived, and observed them prospecting and initiating building. More often, however, nests were found at later stages and initiation dates had to be estimated, for which the following timings were used: 14‐day incubation (beginning on the day the clutch was completed); clutch initiation date was determined by subtracting the number of days equivalent to clutch size −1 from the clutch completion date (assuming one egg laid per day); nest initiation date was 7 days prior to clutch initiation.

After creating the set of male locations for each arriving female, we modelled each male's propensity for being chosen based on the habitat attractiveness at his location (value of *hab*) and his connectivity with respect to other males. We borrow the latter measure from metapopulation theory (Hanski et al., 2017), where connectivity is a property of a habitat patch: it measures, for a given patch, how many other habitat patches are in the vicinity of the focal patch, adjusted for distance (shorter distances give higher connectivity). In our application of this approach, ‘patches’ are males (not habitats), and connectivity offers a way to express the degree to which a focal male is surrounded by other males: the connectivity of male *i* is

$$C_{i}=\sum_{j\neq i}e^{-d_{\mathrm{ij}}},$$

where *d*_*ij*_ is the distance in metres between the focal male *i* and a competitor male *j*. All males simultaneously present in the same study site as male *i* (maximal distance of *c*. 2 km) were considered competitors. The variation in the maximum distance at which males were included as competitors (due to variation in the size of different study sites) does not bias the analysis, because the exponential decay means that in practice faraway males do not ‘count’. We ran the analysis twice: in the main analysis we included the spatial midpoint of two paired playback speakers as a ‘competitor’ male when computing real males' connectivities; in a secondary, alternative analysis, we ignored the playbacks. There was no qualitative difference in the outcome of these analyses.

The analysis uses an information theoretic approach (Akaike, 1974; Burnham & Anderson, 2002) to compare models modified for mate choice (Safari et al., 2019). Due to small sample size (58 females), we use AICc as a criterion (Hurvich & Tsai, 1989). We model male attractiveness ($A_{i}$) as

$$A_{i}=H_{i}^{\alpha_{1}}C_{i}^{\alpha_{2}},$$

where parameters *α*_1_ and *α*_2_ are set to zero in a null model where habitat (H) or male connectivity (C) does not matter and thus $A_{i}=1$ for all males. Note that a playback can contribute to real males' attractiveness scores, but playbacks themselves have no *A*_*i*_ values as females cannot choose a playback as a mate.

There are three models in addition to the null model, in which negative *α* values imply lower attractiveness and positive ones imply higher attractiveness if the underlying variable (either habitat attractiveness or connectivity) increases. Note that the a priori hypothesis for *α*_1_ is that it should be positive: being in attractive habitat improves a male's attractiveness, however, we do not impose a constrain *α*_1_ > 0 but estimate its value based on data. For *α*_2_, a positive estimate would indicate that males benefit from being congregated, thereby supporting the female preference hypothesis. However, since males also compete with one another, high connectivity may simply signal intense competition, and since only one of the many males will succeed to pair with an arriving female, it is also possible that *α*_2_ < 0. If the model produces a combination where *α*_1_ > 0 and *α*_2_ < 0, the interpretation is that males benefit from singing in attractive nesting habitat (since this is where females look for mates as well as nest sites), but that males do so *despite* other males also settling preferentially in attractive habitat, not *because* other males do so.

We thus have four models in total. In the ‘habitat only’ model, *α*_1_ is estimated based on data while *α*_2_ is set to zero. The ‘connectivity only’ model makes the opposite assumptions: *α*_1_ is set to zero while *α*_2_ is estimated based on data, and in the full model, both are simultaneously estimated based on data of actual female choices. The null model estimates neither *α*_1_ nor *α*_2_ but sets them to zero, as explained above.

Note that the values of attractiveness themselves do not have a strictly defined upper bound, but since the female chooses one mate as a male among those present, they will be converted to probabilities which sum up to 1 for each mate choice situation. The probability that male *i* is chosen among all *j* is modelled as $p_{i}=\frac{A_{i}}{\sum_{j}A_{j}}$. In reality, the female chose one of the males, and the log likelihood of this happening is $\ln(p_{i}$). Since, for the three nonnull models, the values of $p_{i}$ depend on *α*_1_ and/or *α*_2_, our method seeks, within each model, the log‐likelihood maximizing values of these parameters (the sum of $\ln(p_{i}$) for all choices made by females). We then compared the four models using AICc (Burnham & Anderson, 2002; Hurvich & Tsai, 1989), using the same criteria for support as in Analysis 1.

As the sum of $p_{i}$ for all males present for one focal female is 1, the model is able to handle the situation that the number of potential mates varies between females without this biasing the procedure. Specifically, all males end up with higher values for their connectivities if more males are added to a situation, but since males are then compared against each other, the overall elevation cancels out and only the relative connectivities matter. This makes the model appropriate for testing whether females are attracted to groups of males to an extent that it makes it beneficial for males to appear in congregations, as suggested by the female preference hypothesis.

## 3 RESULTS

### 3.1 Analysis 1: Testing the habitat detection and habitat choice copying hypotheses

**FIGURE 2:**
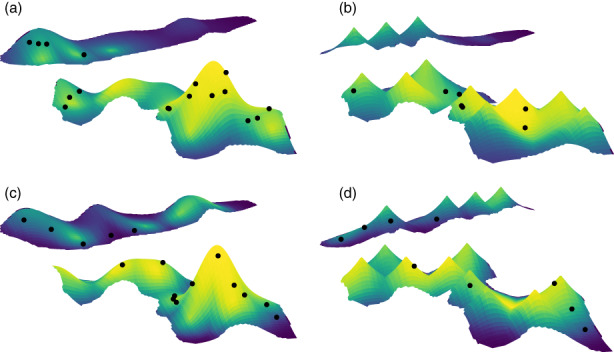
Plots illustrating the effects of spatial variation in habitat attractiveness (*hab*) and proximity to conspecific playbacks of any type (*prox.all*) in 2017 (a and b respectively) and 2018 (c and d respectively) on the density of males in EW, as predicted by the top model (*hab* + *prox.all*). Spatial variation in plot height represents spatial variation in covariate values, with peaks representing either areas of high habitat attractiveness (a and c) or the locations of playback stations (i.e. territory centres of simulated males; b and d). The colour gradient represents spatial variation in male density (increasing from blue to yellow hues). The black dots represent the actual locations of settled males (note that because of the perspective, not all male locations are visible in the plots.

Males settled and bred in all the sites containing conspecific song playbacks, but in varying numbers (Table 2). Models including covariates representing spatial variation in habitat attractiveness (i.e. spatial variation in cumulative nest density) and proximity to conspecific playbacks predicted male density better than the null model of random settlement (Table 3). The best model included habitat attractiveness (variable *hab*) and proximity to conspecific playbacks of any type (variable *prox.all*), followed closely by the model including habitat attractiveness and proximity to conspecific playbacks with low song rates (variable *prox.lc*). Based on the best models including either *prox.all* or *prox.lc*, the unstandardized effect sizes of these variables were, respectively, *β*_prox.all_ = 1.773 (95% CI = 0.057–3.489) and *β*_prox.lc_ = 1.427 (95% CI = −0.032–2.885). Model‐averaged estimates for these parameters (based on all models containing *prox.all* or *prox.lc*) were similar to those from the best model containing each parameter (model‐averaged estimates: *β*_prox.all_ = 1.773, *β*_prox.lc_ = 1.428). The unstandardized effect size of habitat attractiveness was similar in both models with substantial support (best‐supported model: *β*_hab_ = 0.109, 95% CI = 0.050–0.169; second‐best model: *β*_hab_ = 0.105, 95% CI = 0.045–0.165). Whether averaging estimates of *hab* across models with ΔAICc ≤ 2 or across all models containing *hab*, *β*_hab_ = 0.107. Figure 2 illustrates the positive relationship between predicted male densities and habitat attractiveness in EW in 2017 and 2018 (Figure 2a,c respectively), and the positive relationship between predicted male densities and proximity to conspecific playbacks of any type (Figure 2b,d respectively).

**TABLE 2:** Settlement of male and female Wood Warblers in experimental sites containing conspecific song playbacks in 2017 and 2018

Site	2017		2018	
	Total males	Total females	Total males	Total females
EW	22	9	21	7
LB	1	1	1	1
HB	NA	NA	5	4

**TABLE 3:** Model comparison table for point process models relating male density to spatial variation in habitat attractiveness and proximity to conspecific song playbacks. Each point pattern represented the cumulative settlement locations of male Wood Warblers within a given site and year (see text for details)

Model^a^	AICc	ΔAICc	*w*_*i*_
*hab + prox.all*	−162.60	0.00	0.53
*hab + prox.lc*	−162.02	0.58	0.39
*hab*	−159.01	3.86	0.08
*prox.lc*	−149.67	13.20	<0.01
*prox.all*	−149.42	13.44	<0.01
*null*	−141.59	21.44	<0.01

^a^ Explanatory variables: *hab* = habitat attractiveness, represented by kernel‐estimated nest density; *prox.all* = proximity to simulated territory (i.e. song playback) of any type of male, represented by the following distance decay function: exp(−distance/200 m); *prox.lc* = proximity to simulated territory (i.e. song playback) of male with low competitive ability, represented by the following distance decay function: exp(−distance/200 m).

Across the entire range of proximity values to conspecific playbacks of all types and conspecific playbacks with low song rate within a study site, the density of males varied by a factor of 5.804 and 4.145 respectively. Across the entire range of habitat attractiveness values, the density of males varied by a factor of 12.894.

### 3.2 Analysis 2: Testing the female preference hypothesis

Based on a total of 59 settlement choices made by 50 individual females, we found that the best predictor of male pairing success was habitat attractiveness (Table 4). There are two well‐supported models: the best model included habitat attractiveness only, followed by a model that had habitat attractiveness and connectivity as explanatory variables. The effect of habitat attractiveness on male pairing success (*α*_1_) was positive in both models (*hab* only: 0.625 regardless of whether we include or exclude playbacks, *hab* + *connectivity*: 0.653 regardless of including or excluding playbacks). In contrast, the effect of connectivity on male pairing success (*α*_2_) was consistently negative (*hab* + *connectivity*: −0.059 when including and −0.063 when excluding playbacks, *connectivity* only: −0.043 when including and −0.052 when excluding playbacks).

**TABLE 4:** Model comparison table for female mate choice models. The main analysis is given first, with the brackets indicating an alternative version that does not make use of playbacks contributing to male attractiveness (i.e. in brackets we use an assumption that females completely ignore playbacks)

Model^a^	AICc	ΔAICc	*w*_*i*_
*hab*	243.01 (243.01)	0 (0)	0.57 (0.50)
*hab + connectivity*	243.95 (243.51)	0.94 (0.50)	0.36 (0.43)
*null*	248.10 (248.10)	5.09 (5.09)	0.04 (0.04)
*connectivity*	249.50 (249.05)	6.49 (6.03)	0.02 (0.03)

^a^ Explanatory variables: *hab* = habitat attractiveness (represented by kernel‐estimated nest density), measured at each male's location; *connectivity* = measure of the degree to which each male is surrounded by other males (see text for details).

Based on the best model (*α*_1_ = 0.625 whether we include or exclude playbacks), a male's chances of attracting a female increase by 6.1% with each 10% increase in habitat attractiveness (1.1^0.625^ = 1.061). The second best model, which is the only well‐supported model that provides an estimate for the effect of connectivity, predicts that a male's chance of attracting a female declines by approximately 0.6% with each 10% increase in connectivity (1.1−^0.06^ = 0.994). These conclusions remain qualitatively unchanged if we perform model averaging; the percentages 6.1% and 0.6% change to 6.3% (6.3%) and 0.2% (0.3%), respectively, if we average over the two models with good support (numbers in brackets exclude playbacks), and to 5.8% (5.8%) and 0.2% (0.3%), respectively, if we average over all models (with little weight on poorly performing models, as is appropriate).

## 4 DISCUSSION

We tested predictions of three hypotheses for conspecific attraction among males of a nomadic songbird: the habitat detection hypothesis, the habitat choice copying hypothesis and the female preference hypothesis. We conclude that the habitat detection hypothesis is the most likely explanation for conspecific attraction and aggregation in male Wood Warblers, based on the following results: (1) males were attracted to conspecific song playbacks, but fine‐scale heterogeneity in habitat attractiveness was the better predictor of spatial patterns in the density of settling males; (2) male pairing success did not increase, but instead slightly decreased, as connectivity with other males (i.e. the number and proximity of neighbouring males) increased.

While it would be premature to conclude that conspecific presence is irrelevant for habitat evaluation, our first result suggests that decisions of previous settlers do not override what a male sees himself. By extension, males probably do not settle near conspecifics because doing so increases their chances of correctly identifying good habitat—they can and do find good habitat (i.e. habitat that is attractive to females) in the absence of any social information. Although a few studies have invoked reliance on social information to explain incongruities between settlement or breeding investment choices and apparent habitat quality (Betts et al., 2008; Forsman et al., 2008; Nocera et al., 2009), empirical evidence suggests that animals rarely discount their own (nonsocial) information about the environment (Rieucau & Giraldeau, 2011). Furthermore, as habitat selection is a hierarchical process in space and time, animals are not constrained to use a single source of information at all scales (Lima & Zollner, 1996; Mayor et al., 2015). Cues of conspecific (or heterospecific) presence that can be detected at relatively long distances (e.g. vocalizations or pheromones) are especially likely to be relevant during the search phase of habitat selection, as they may be perceived well‐before many habitat features can be seen in detail (Childress & Herrnkind, 2001; Fletcher, 2006). Indeed, the results of our own and other playback experiments in birds (Albrecht‐Mallinger & Bulluck, 2016; Cornell & Donovan, 2010; Mann et al., 2021; Rushing et al., 2015) and frogs (James et al., 2015) suggest a sequential use of social information and direct habitat assessment.

Our second analysis demonstrated that male Wood Warblers do not attain postsettlement mating benefits as a consequence of their proximity to one another. If anything, our data suggest that male pairing success is reduced when other males are nearby. Although we are aware of one study in Wood Warblers (Herremans, 1993) and one study in Least Flycatchers *Empidonax minimus* (Tarof et al., 2005) that reported a female preference for aggregated males, several studies have found no evidence that a male songbird's success in attracting a female (or in siring extra‐pair offspring) is a function of his spatial position relative to other males (Grendelmeier et al., 2017; Manica et al., 2020; Safran, 2007; Winnicki et al., 2019). The contrasting results of our study and the study of Herremans (1993) are likely due at least in part to the challenge of defining suitable habitat. Herremans (1993) determined that females preferred aggregated males based on two results: (1) the proportion of paired males increased as nearest neighbour distances decreased; (2) the proportion of paired males increased as male density increased. These patterns could be explained equally well by a concentration of males in preferred habitat, but Herremans (1993) excluded this as a possible explanation based on the fact that aggregations were not aligned with spatial variation in caterpillar abundance (Cramp, 1992). While the importance of food availability is indisputable, it may not be the primary criterion upon which breeding habitat selection is based, especially given that Wood Warblers show considerable dietary flexibility (Mallord et al., 2017; Maziarz & Wesołowski, 2010). Nonetheless, Herremans (1993) concluded that males aggregate to attain social benefits after finding a low concordance between territory/nest density and this particular habitat feature.

Weak or no support for the female preference hypothesis is perhaps less surprising when considered from the perspective of the process determining male mating success: mate choice by females. If indirect genetic benefits have appreciable effects on increasing female fitness, females should invest in phenotypic comparison of males, which renders male aggregation to reduce the cost of this process plausible. Therefore, as applied to species where a female's choice of mate(s) is coupled with or constrained by her choice of nest site, the female preference hypothesis assumes indirect genetic benefits are important enough determinants of female fitness that the opportunity to efficiently compare potential social and/or extra‐pair mates is a key factor in female settlement decisions. However, multiple reviews of theory and empirical studies (Akçay & Roughgarden, 2007; Kirkpatrick & Ryan, 1991; Kokko et al., 2003; Møller & Alatalo, 1999) argue that effects of indirect genetic benefits on female fitness are typically small, particularly relative to factors influencing a female's own survival (Achorn & Rosenthal, 2020). Corroborating this result, experiments in Pied Flycatchers *Ficedula hypoleuca* (Alatalo et al., 1986), House Wrens *Troglodytes aedon* (Eckerle & Thompson, 2006) and Barn Swallows *Hirundo rustica* (Safran, 2007) have shown that females heed nesting resources more than male characteristics when making settlement decisions. Taken together, these studies indicate that a reduction in mate assessment costs is likely to be of negligible importance when females choose mates and habitat concurrently.

There are two additional aspects of Wood Warbler breeding ecology that further support the habitat detection hypothesis as a probable explanation for male conspecific attraction in this species. First, the frequent within‐season and between‐season movements of male Wood Warblers to new territories (Herremans, 1993; Luepold, 2022; Temrin et al., 1984) suggest that the benefit of increased search efficiency is likely to be especially relevant. Frequent movements also render Allee effects that require a relatively long lag time to be realized (e.g. protection of offspring against predation) improbable as drivers of conspecific attraction because the social conditions at settlement are almost certainly not representative of the conditions that will be present by the time there are young to protect. Second, nest predation in this species often occurs at night and by predators that Wood Warblers cannot defend against (Grendelmeier et al., 2018; Maag et al., 2022; Maziarz et al., 2019), which makes the putative anti‐predation benefits of aggregation (i.e. group vigilance or defence) biologically unlikely.

It has been reported that some migrant songbirds (including Wood Warblers) exhibit heterospecific attraction to species with earlier arrival to breeding areas (e.g. Forsman et al., 2007; Szymkowiak et al., 2017; Thomson et al., 2003). These studies assume that because individuals of earlier‐arriving species have been in the breeding area longer, they know more about it and are therefore worth copying (Seppänen & Forsman, 2007). We can presently only speculate as to whether Wood Warblers in our study sites exhibit heterospecific attraction, but to the extent that they do, it is probably due to the same process that appears to underlie conspecific attraction—namely, increased detection probability (causing attraction to a general area) rather than habitat choice copying per se. This is because no resident or short‐distance migrant songbirds breeding in our study forests reliably favour the same nesting habitat as Wood Warblers (S. Luepold, pers. obs.). Thus, copying the habitat choices of these putatively ‘more informed’ species would often land male Wood Warblers in places where female Wood Warblers are unlikely to nest.

We argue here that because male Wood Warblers appear capable of identifying attractive nesting habitat with high precision, and because they do not obtain proximity‐based mating benefits, conspecific attraction is primarily a consequence of increased detection probability. We acknowledge, however, that detection probability alone may not fully explain a male's choice to sing 50 m from a conspecific when there is comparable habitat available 100 m or more away. Given the aforementioned mobility of male Wood Warblers within and between forest patches, it seems indeed unlikely that a male would be ignorant of the option of being (at least somewhat) farther away. As a post‐hoc explanation, we therefore suggest that such cases of apparently ‘unnecessary’ proximity may be related to how priority of access to a space (and to females settling within it) is achieved. As bounded spatial entities, territories emerge out of behavioural interactions with competitors (Huxley, 1934; Morrell & Kokko, 2005; Potts & Lewis, 2014; Stamps & Krishnan, 1995). Female Wood Warblers sometimes wander widely when searching for nest sites (Cramp, 1992), so it behoves a male to exclude other males from as large an area as possible. To accomplish this, a newly arrived male can attempt to evict a resident conspecific outright, or win space via engaging the resident in frequent but relatively low‐cost aggressive interactions (e.g. chases; Ezaki, 1995; Stamps & Krishnan, 1995; Stamps & Krishnan, 2001). Anecdotal evidence from our playback experiment is consistent with this explanation, as we regularly observed males singing directly above the speakers, or even physically attacking them.

In conclusion, while male Wood Warblers are attracted to conspecifics, they do not prioritize social information when making settlement decisions, nor do they obtain postsettlement mate attraction benefits from their proximity to one another. Rather, it seems that conspecific attraction is likely a consequence of using social information to locate potentially suitable habitat and/or the attempt to claim all or part of the area occupied by a conspecific. Such a habitat selection strategy may allow males to minimize search time, while avoiding being duped into settling in locations where the prospects of attracting a mate are poor. However, our results also suggest that when this strategy results in high local densities of males, the benefits of being in good (i.e. attractive) nesting habitat are at least partially offset by increased competition for mates.

## AUTHOR CONTRIBUTIONS

Shannon B. Luepold and Gilberto Pasinelli conceived the study and designed the playback experiment; Hanna Kokko conceived the idea to use metapopulation theory to quantify effects of male aggregation on mating success; Shannon B. Luepold and Alex Grendelmeier collected the data; Shannon B. Luepold and Hanna Kokko analysed the data; Shannon B. Luepold led the writing of the manuscript. All authors provided critical input on drafts and gave approval for publication.

## CONFLICT OF INTEREST

The authors declare no conflict of interest.

## Supporting information


Appendix S1

## Data Availability

All data files are available from the data repository of the Swiss Ornithological Institute https://doi.org/10.5281/zenodo.7123330 (Luepold et al., 2022).
