# Supplementary material for: Habitat detection, habitat choice copying or mating benefits: What drives conspecific attraction in a nomadic songbird?
Source: J Anim Ecol. 2022 Nov 22;92(1):195–206. doi: 10.1111/1365-2656.13844 (PMC10100052; doi:10.1111/1365-2656.13844)
Supplement: Supplementary file 1 — Appendix S1 [file JANE-92-195-s001.pdf]

Supporting Information: Habitat detection, habitat choice copying or mating benefits: what drives conspecific attraction in a nomadic songbird?

S1. Details of data collection for territory locations of marked males 2017-19

We captured most males using mist nets (Ecotone, Gdynia, PL) and playback of conspecific song (n=127). A few males were captured while provisioning nestlings (n=6). We captured females with a dipnet on the nest during incubation (n=54) or with a mist net while feeding nestlings (n=8). In addition to being marked with 3 plastic color rings (2.1 – 2.3 mm) and a numbered aluminum ring, most males in 2017 and 2018 (93%, n=42 and 56%, n=50, respectively) also received a small (0.3 g) radio transmitter (Holohil, Carp, ON, CAN). The transmitter was mounted on the bird using a leg-loop harness (Rappole & Tipton 1991).

In 2017, when almost all captured males were fitted with a radio transmitter, we did 2-hour telemetry sessions with a given individual and marked a singing location point every three minutes. During the 2017 field season, we observed that male movements within a given telemetry session were generally limited in spatial extent (e.g, within an area ≤ 0.5 ha). Between sessions (i.e., on different days), however, singing locations could shift substantially (> 1 km). Secondly, turnover rate within the study areas was high throughout the breeding season, with the locations and identities of birds present in a given area shifting regularly. We therefore adjusted our method for collecting location data on color-ringed birds (with and without transmitters) in 2018 and 2019 by reducing the amount of time we spent with a given individual from two hours to 15 minutes. This approach yielded five location points per individual per observation (one point every 3 minutes), which still allowed us to delineate a daily "territory" while also covering more of the study area on a given visit.

In addition to recording locations during telemetry sessions (2017) and surveys (2017-2019), we also opportunistically recorded locations of marked individuals while doing other

fieldwork (e.g., nest checks, playback maintenance, etc.). We then averaged the locations of a given male on each day he was observed to obtain a single territory location per day for each male. All nests where a male was known to be the social father were included as his (inferred) territory location on the day the nest was initiated.

Table S1. Summary information for experimental sites.

Site	Area (ha)	Number of playbacks/ha		Furthest possible distance from any playback (m)	
		2017	2018	2017	2018
EW	138.61	0.08	0.09	1081.96	494.90
LB	50.90	0.10	0.08	321.63	571.39
HB	57.81	-	0.05	-	933.19

Table S2. Song rate (low=3 trills min^{-1}, high=6 trills min^{-1}) and nearest neighbor distance for each playback in 2017.

Playback ID	Song rate	Nearest neighbor distance (m)
EW01	low	260.63
EW03	high	253.93
EW06	low	253.93
EW07	high	254.53
EW08	low	253.32
EW10	low	249.29
EW12	high	249.29
EW13	low	252.44
EW15	low	254.66
EW16	low	254.66
EW17	high	261.52
LB01	high	250.46
LB02	high	250.46
LB04	high	508.70
LB06	low	253.33
LB07	low	253.33

Table S3. Song rate (low=3 trills min^{-1}, high=6 trills min^{-1}) and nearest neighbor distance for each playback in 2018.

Playback ID	Song rate	Nearest Neighbor Distance (m)
EW01	high	251.07
EW02	low	251.07
EW04	high	261.04
EW05	low	253.95
EW06	high	253.95
EW07	low	254.49
EW08	high	257.01
EW09	low	250.59
EW12	low	250.59
EW16	high	503.24
EW18	low	250.55
EW19	high	250.55
EW20	low	250.96
LB02	low	253.87
LB03	high	253.87
LB05	low	255.38
LB06	high	255.38
HB01	high	772.27
HB02	low	501.60
HB04	high	501.60

S2. Creating the covariate "hab" based on cumulative nest densities

We used spatial variation in the cumulative density of nests within a site between 2010 and 2020 to represent spatial variation in habitat features that are attractive to females (*hab*). To ensure that hotspots of elevated nest density were not the result of females renesting in close proximity to their previous failed nesting attempt, we filtered nest location data based on the following two criteria: 1) for marked females, we excluded known renests within 200 m of their previous nest; 2) if multiple nests within 200 m of one another were from unmarked females, only those that were active simultaneously (i.e., certainly from different individuals) were retained in the analysis. This resulted in a cumulative total of 299 nests, with a maximum of 74 nests per site. While a clear majority (91%) of these points were actual nests, in a few cases (n=26) a pair (male + female) was observed at that point but the precise nest location was not found. Due to varying research foci and logistical constraints between seasons, not all sites were monitored in all years. The minimum number of years with data for a given site was four, the maximum ten (Table S4).

After creating a point pattern of nest locations for each site, we used kernel density estimation implemented in spatstat (Baddeley et al., 2015) in R version 3.6.3 (R Core Team, 2020) to generate a 10×10 m continuous raster of nest density within a site. We used a Gaussian kernel and a smoothing bandwidth of 100 m, which corresponds to the diameter of a typical Wood Warbler territory (Cramp, 1992). For more intuitive interpretation, densities were scaled to nests/km^2 (Fig. S1A). We corrected for differences in density resulting from variable sampling effort by dividing density estimates by the number of years data was collected in a given site. It is possible that some nesting "hotspots" were not represented in the density maps from sites with relatively few years of data. This could potentially result in the apparent relationship between male settlement patterns and spatial variation in habitat attractiveness being weaker than it actually is.

Spatial consistency in territory and/or nest locations across years is not an artefact of individual site fidelity but instead reflects the decisions of multiple settlers (individual return rates are <5% in our study sites and in other regions, e.g., Herremans, 1993; Wesołowski et al., 2009). Interannual spatial consistency in settlement locations is most likely due to underlying habitat characteristics rather than social information collected in previous years for the following proximate and ultimate reasons: (1) The observation of reproductively successful conspecifics (e.g., adults with fledglings) does not necessarily provide information about the habitat in which the fledglings were produced, because Wood Warbler fledglings move away from the nest area relatively quickly (e.g., 100 m away the day after leaving the nest, ca. 500 m away a week after leaving the nest, Cramp 1992). Thus, prospectors would still have to rely on other means (presumably direct assessment of habitat features) to identify potential nesting habitat within that general area. (2) As a consequence of the low site fidelity exhibited by Wood Warblers, many locations are not occupied every year. This means that individuals often settle in a territory in year *t* for which there was no social information available in year *t-1*, *t-2*, etc. (3) Choosing habitat in year *t* based on the breeding success of others in year *t-1* is an evolutionarily favored strategy when reproductive outcomes are correlated in space and time (Doligez et al., 2003). These circumstances do not apply in our system, as nests with different fates are not spatially segregated (Table S5).

Table S4. Number of Wood Warbler nests per site per year. “NA” indicates the site was not monitored that year. Counts exclude renests of the same female within 200 m (see text).

	Site						
Year	EW	HB	KL	LB	LW	MS	SP
2010	NA	NA	5	4	5	3	14
2011	NA	4	6	2	10	6	4
2012	4	1	7	NA	0	4	4
2013	12*	1	6	1	3	7	3
2014	6*	NA	8	NA	13	7	10
2015	NA	NA	7	NA	11	5	8
2017	10*	NA	8	1*	NA	1	NA
2018	8*	5*	10	1*	5	4	NA
2019	3	NA	10	NA	3	2	11
2020	6	NA	6	NA	NA	0	13
Total	49	11	74	9	50	39	67

*Song playbacks simulating conspecifics were present.

Table S5. Results of tests of spatial segregation between successful and failed Wood Warbler nests at seven sites in the Swiss Jura.

Study Site	Years of data	Total nests (fledged, failed)	*T**	P-value
EW	7	48 (21, 27)	0.069	0.42
HB	4	12 (5, 7)	0.209	0.23
KL	10	67 (35, 32)	0.034	0.93
LB	5	10 (3, 7)	0.221	0.42
LW	8	51 (16, 35)	0.087	0.51
MS	10	39 (17, 22)	0.052	0.72
SP	8	54 (33, 21)	0.091	0.39

* Test statistic measuring constancy of the spatial distribution of the different point types (i.e., fledged and failed nests).

S3. Creating the covariates representing proximity to conspecific playbacks (prox.all and prox.lc)

To create the covariates representing proximity to a simulated male of any type and proximity to a male of low competitive ability, we used a multi-step approach. We first used the *distmap* function in spatstat to generate a 10x10 m raster with pixel values equal to the distance to the nearest simulated conspecific of any type, or of low competitive ability, respectively. Because the response of animals to a given feature in the environment often decays exponentially with distance (Lazenby et al. 2021; Whittington et al. 2011), we then transformed pixel values with linear distances to a measure of proximity using the formula $e^{-d/\alpha}$, where d is the linear distance to a feature and α is the distance threshold at which values approach 0. This transformation scales proximity values to a range between 0 and 1, with values near 1 being close to a feature and values near 0 being far away. Note that with this transformation, a positive value of $\beta_{prox.all}$ or $\beta_{prox.lc}$ indicates that males are attracted to settle near simulated conspecifics of any type or of low competitive ability, respectively.

We then compared the AICcs of models with α's ranging from 100 – 500 m to determine which proximity measure best predicted male settlement density. We found that the effect of proximity to simulated conspecifics as well as the effect of proximity to simulated less competitive conspecifics on male settlement density was best described by exponential decay at 200 m (i.e., $e^{-d/200}$), though all exponential decay variables were comparable (i.e., $\Delta AICc \leq 2$, Tables S6 and S7). Therefore, we used the covariate calculated by $e^{-d/200}$ to represent the effects of proximity to social cues (*prox.all* or *prox.lc*, Fig. S1B and S1C) in the candidate set of models for testing the habitat detection hypothesis and the habitat choice copying hypothesis. The above analyses were conducted in R version 3.6.3 (R Core Team, 2020).

Table S6. Model selection table for point process models relating male density to different variables representing proximity to song playbacks of any type. The best-supported variable (*prox.all.200*) was included in subsequent models with the name "*prox.all*."

Model[a]	AICc	ΔAICc	w_i
prox.all.200	-149.423	0.000	0.281
prox.all.300	-148.812	0.610	0.208
prox.all.100	-148.801	0.623	0.206
prox.all.400	-148.214	1.209	0.154
prox.all.500	-147.739	1.685	0.121
lin.dist	-144.934	4.490	0.030

[a] Explanatory variables: *lin.dist* = linear distance to nearest playback of any type; *prox.all.100* = exp(-distance/100 m); *prox.all.200* = exp(-distance/200 m); *prox.all.300* = exp(-distance/300 m); *prox.all.400* = exp(-distance/400 m); *prox.all.500* = exp(-distance/500 m).

Table S7. Model selection table for point process models relating male density to different variables representing proximity to playbacks with low song rates. The best-supported variable (*prox.lc.200*) was included in subsequent models with the name "*prox.lc*."

Model[a]	AICc	ΔAICc	w_i
prox.lc.200	-149.344	0.000	0.283
prox.lc.100	-148.903	0.441	0.227
prox.lc.300	-148.607	0.737	0.195
prox.lc.400	-148.339	1.357	0.143
prox.lc.500	-147.534	1.812	0.114
lin.lc.dist	-145.311	4.034	0.038

[a] Explanatory variables: *lin.lc.dist* = linear distance to nearest playback with low song rate; *prox.lc.100* = exp(-distance/100 m); *prox.lc.200* = exp(-distance/200 m); *prox.lc.300* = exp(-distance/300 m); *prox.lc.400* = exp(-distance/400 m); *prox.lc.500* = exp(-distance/500 m).

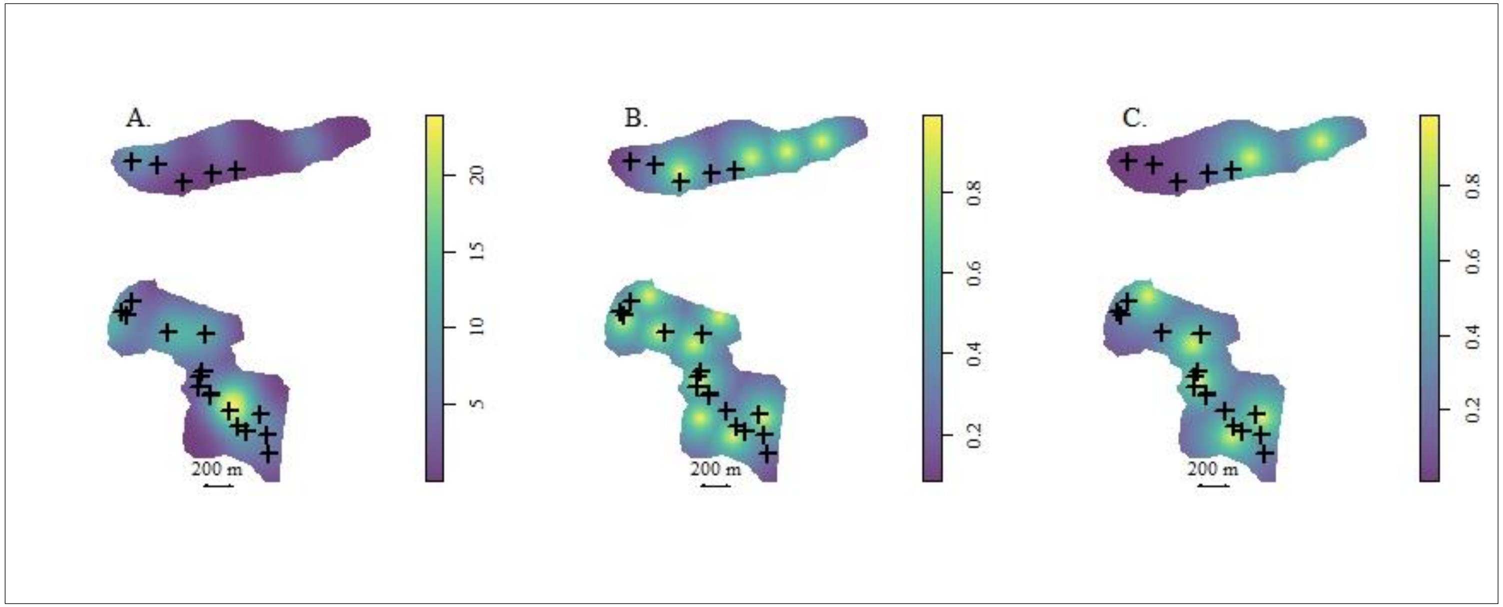

Figure S1. Illustration of male settlement locations (black crosses) in EW in 2018 in relation spatial covariates representing habitat attractiveness (A), proximity to playbacks of any type (B) and proximity to playbacks with low song rates (C). Spatial variation in habitat attractiveness (A) corresponds to variation in cumulative kernel-estimated nest density (nests/km^2). Proximity covariates (B and C) were calculated using the following exponential decay function ($e^{-d/200}$), which scales distance values to be between 1 (near the simulated territory) and 0 (far away from the simulated territory).

S4. Quadrature scheme used in point process models in Analysis 1

Quadrature schemes are required by point process models to estimate the model pseudolikelihood (Baddeley et al. 2015). As described in the user manual for the R package *spatstat*, a quadrature scheme consists of the original data point pattern, an additional pattern of dummy points, and a vector of quadrature weights for all these points. We used the default quadrature scheme in spatstat for generating the pattern of dummy points, which results in a regular rectangular grid. The frame containing the observation window (in our case the polygon representing a study site) is divided into an array of *d*-by-*d* tiles, and one dummy point is generated at random within each tile. If the observation window is not rectangular (as in our case) any dummy points lying outside the window are deleted. By default, the *d* x *d* dimensions of the grid of dummy points are determined by the number of points in the data point pattern. For data patterns with up to 225 points, the minimum number of dummy points required by the ppm function in spatstat is a 32 x 32 grid. We used this default setting for generating dummy points (see Table S7 for the number and spacing of dummy points for each point pattern included in Analysis 1). The weight of each quadrature point (i.e., each data and dummy point) is calculated by dividing the area of the tile by the number quadrature (data + dummy) points.

Table S7. Description of quadrature scheme used in point process model for Analysis 1.

Point Pattern	Number of Quadrature Points (data, dummy)	Dummy Point Spacing (in m)[a]	Total Weight[b]
EW in 2017	389 (22, 367)	56.8 x 78.3	1.388
LB in 2017	350 (1, 349)	58.7 x 28.4	0.509
EW in 2018	388 (21, 367)	56.8 x 78.3	1.388
LB in 2018	350 (1, 349)	58.7 x 28.4	0.509
HB in 2018	429 (5, 424)	75.5 x 25.5	0.578

[a] Spatial dimensions of the dummy point grid (horizontal x vertical)

[b] Sum of the weights of all quadrature points

LITERATURE CITED

Baddeley, A., Ruback, E., & Turner, R. (2015). *Spatial point patterns: methodology and applications with R*. London: Chapman and Hall/CRC Press.

Cramp, S. (1992). *The birds of the western Palearctic* (Vol. 6): Oxford University Press.

Doligez B., Cadet C., Danchin E., Boulinier T. (2003). When to use public information for breeding habitat selection? The role of environmental predictability and density dependence. *Animal Behavior, 66*, 973–988. https://doi.org/10.1006/anbe.2002.2270

Herremans, M. (1993). Clustering of territories in the Wood Warbler *Phylloscopus sibilatrix*. *Bird Study, 40(1)*, 12–23. https://doi.org/10.1080/00063659309477124

Lazenby, K. D., Coates, P. S., O'Neil, S. T., Kohl, M. T., & Dahlgren, D. K. (2021). Nesting, brood rearing, and summer habitat selection by translocated greater sage-grouse in North Dakota, USA. *Ecology and Evolution, 11(6)*, 2741–2760. https://doi.org/10.1002/ece3.7228

R Core Team (2020). R: A language and environment for statistical computing. R Foundation for Statistical Computing, Vienna, Austria. https://www.R-project.org/.

Rappole, J. H., & Tipton, A. R. (1991). New harness design for attachment of radio transmitters to small Passerines. *Journal of Field Ornithology*, *62*, 335-337.

Wesołowski, T., Rowiński, P., & Maziarz, M. (2009). Wood Warbler *Phylloscopus sibilatrix*: A nomadic insectivore in search of safe breeding grounds? *Bird Study, 56(1)*, 26–33. https://doi.org/10.1080/00063650802681540

Whittington, J., Hebblewhite, M., DeCesare, N. J., Neufeld, L., Bradley, M., Wilmshurst, J., & Musiani, M. (2011). Caribou encounters with wolves increase near roads and trails: a time-to-event approach. *Journal of Applied Ecology, 48(6)*, 1535–1542. https://doi.org/10.1111/j.1365-2664.2011.02043.x
